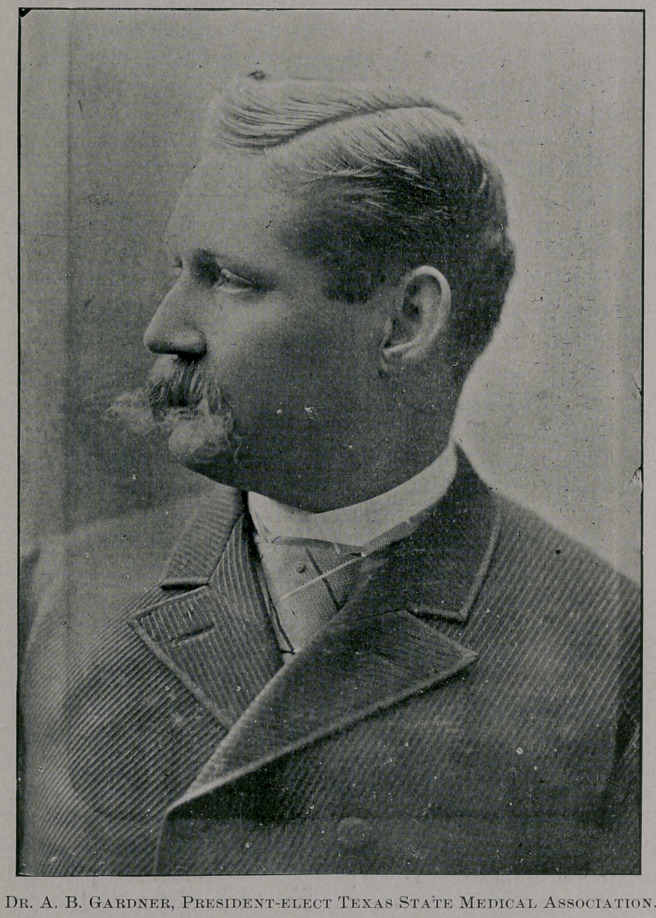# Dr. A. B. Gardner, Bellville, Texas

**Published:** 1899-05

**Authors:** 


### Dr. A. B. Gardner, Bellville, Texas.

President Texas State Medical Association.

Dr. A. B. Gardner, whose portrait we give herewith, the new President of the Texas State Medical Association, elected at the recent meeting at San Antonio, is one of the most popular of the younger members of the profession, and has ever been one of the most enthusiastic working members of the Association. For twenty years he has scarcely failed to attend a meeting, no matter how distant from his home. He has fairly earned the distinguished honor recently conferred upon him. He served eight years on the Judicial Council, and that, too, at the stormiest periods of the society's existence, and it has passed through some cyclones that nearly wrecked it. He has served in every capacity in which he has been called upon to act, and has done it well. That his administration will be creditable and efficient, is certain. It affords us pleasure to add his picture to our gallery of distinguished Presidents; we have published the portraits of all but three, I think, in the last fifteen years.

Dr. Gardner was born in Warren county, Kentucky, November 7, 1852, and is therefore in his forty-seventh year. He received the best schooling to be had in that section in the troublesome times of the Civil war. In 1868 he entered the State University at Lexing-

DR. A. B. GARDNER, PRESIDENT-ELECT TEXAS STATE MEDICAL ASSOCIATION.

ton, Kentucky. Began the study of medicine in 1871, and graduated from the Medical Department, University of Louisville, in 1874. Upon receiving his diploma he removed at once to Texas, locating at the town of McDade, in Bastrop county, and practiced there six years. He then took a full course at Bellevue Hospital Medical College, New York, in 1881, receiving a diploma from that institution. When he returned to Texas he removed to Bellville, where he has resided ever since. He has a fine practice, and is extensively known and universally liked for his excellent social qualities, and is held in high esteem by his colleagues for his professional ability and strictly ethical character.

Dr. Gardner was married in 1876, to Miss Hattie Campbell, of Bastrop, Texas, and they have two children; a son, now at the Texas A. and M. College, and a daughter, Lula E. Gardner, both about grown.

## Figures and Tables

**Figure f1:**